# Merkel Cell Carcinoma on the Face: Case Report

**DOI:** 10.2196/56658

**Published:** 2024-04-08

**Authors:** Shaikha Salah Alhaj, Fatma Abdulghaffar Qaderi, Tarek Ibrahim, Maha Almohammad

**Affiliations:** 1 Mohammed Bin Rashid University of Medicine and Health Sciences Dubai United Arab Emirates; 2 Dermatology Dubai Academic Healthcare Corporation Dubai United Arab Emirates

**Keywords:** carcinoma, metastasis, lesion, biopsy, lesions, skin, Merkel, dermatology, nodules, cancer, oncology, lab, WBC: white blood cell, platelets, dermis, tumor, immunology, histology, histopathology, histopathological, immunological, immunohistochemistry

## Abstract

Merkel cell carcinoma (MCC) is a rare primary neuroendocrine skin tumor that presents as a flesh-colored or bluish-red nodule on the face, neck, or head. Long-term ultraviolet radiation exposure and Merkel cell polyomavirus are associated with MCC pathogenesis. We present a case of MCC on the right cheek in a male patient aged 87 years. Our primary goal in presenting the case is to bring MCC, which is a diagnostic challenge, to the notice of dermatologists and oncologists, as early detection and prompt treatment are important. The patient had a significant past medical history, including diabetes mellitus, hypertension, dyslipidemia, stage 3 chronic kidney disease, benign prostatic hyperplasia, chronic hyponatremia, acute pancreatitis, essential thrombocytosis on hydroxyurea, and ischemic heart disease. The patient presented with a mildly swollen right upper lip showing a poorly defined, relatively homogeneous subcutaneous lesion with a history of persistence for 1.5 months. The clinical examination revealed a 5 × 3–cm nodular lesion on the right side of the cheek with swelling of the right upper lip. Immunohistochemistry markers and histopathological features confirmed the diagnosis of MCC. The patient was referred to the oncology department for further management. MCC of the skin is an aggressive lesion with a high risk of metastasis and recurrence, which is more common in immunocompromised people. Prompt management and treatment of MCC is essential because if left untreated, it can spread to other parts of the body and can also metastasize to lymph nodes and other organs. The patient is 87 years old and has a significant past medical history of diabetes mellitus, hypertension, dyslipidemia, chronic kidney disease stage 3, benign prostatic hyperplasia, chronic hyponatremia, acute pancreatitis, essential thrombocytosis on hydroxyurea, and ischemic heart disease. Currently, the patient presented with a mildly swollen right upper lip showing a poorly defined, relatively homogenous subcutaneous lesion with a history of persistence for 1.5 months. The clinical examination revealed a 5x3 cm nodular lesion on the right side of the cheek with swelling of the right upper lip. Immunohistochemistry markers results and histopathological features confirmed the diagnosis of Merkel cell carcinoma. The patient was referred to the oncology department for further management. Merkel cell carcinoma of the skin is an aggressive lesion with a high risk of metastasis and recurrence, which is more common in immunocompromised people. Prompt management and treatment of Merkel cell carcinoma is essential because if left untreated, it can spread to other parts of the body and can also metastasize to lymph nodes and other organs.

## Introduction

Merkel cell carcinoma (MCC) is a rare primary neuroendocrine skin tumor [1] that usually presents as a flesh-colored or bluish-red nodule on the face, neck, or head [2]. It primarily affects White men older than 65 years and immunocompromised people [3]. Long-term ultraviolet (UV) radiation exposure and Merkel cell polyomavirus are associated with MCC pathogenesis. MCC patients often appear with a quickly developing, painless, hard, glossy, flesh-colored, or bluish-red intracutaneous nodule [4]. Here, we present a case of MCC in a male patient aged 87 years with a mildly swollen right upper lip showing a poorly defined, relatively homogeneous subcutaneous area with a history of persistence for 1.5 months.

## Ethical Considerations

Ethical consent was obtained from the patient before reporting the case for using the patient’s images and clinical information in this paper. The patient understands that his name and initials will not be published and due efforts will be made to conceal his identity.

## Case Report

**Figure 1:**
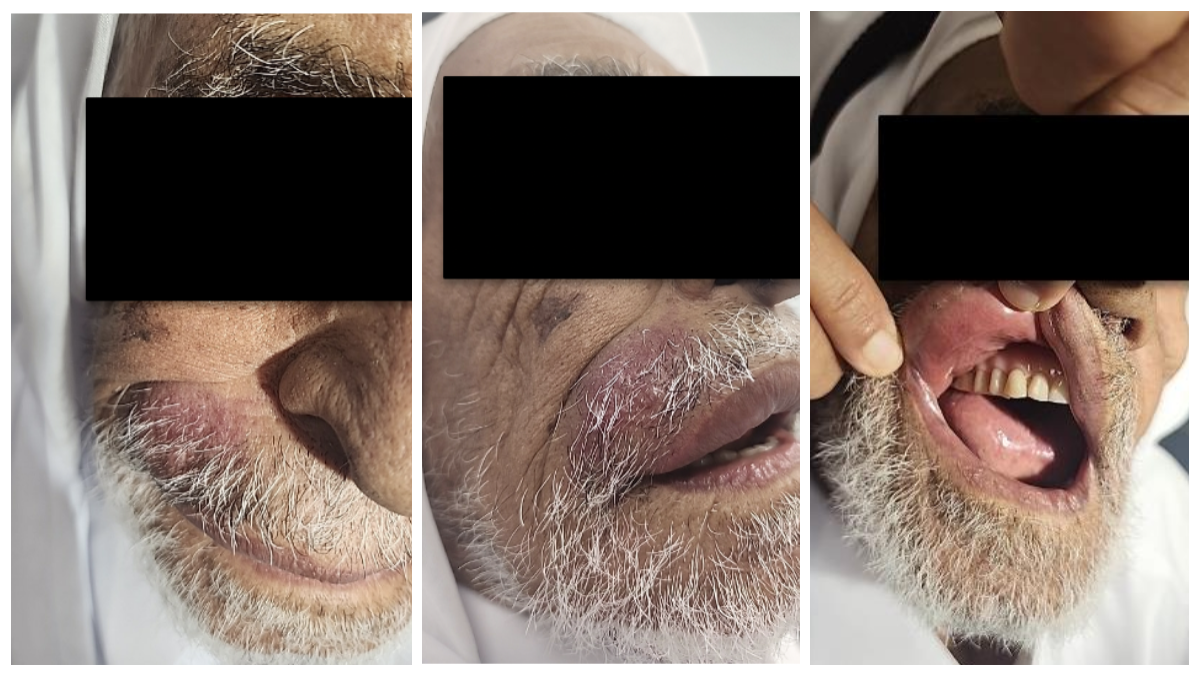
Erythematous plaque on the right upper lip extending to the nasolabial fold. No lesions were seen on the oral mucosal surface.

The patient was aged 87 years and had a past medical history of diabetes mellitus, hypertension, dyslipidemia, stage 3 chronic kidney disease, benign prostatic hyperplasia, diabetic neuropathy, chronic hyponatremia, acute pancreatitis, essential thrombocytosis on hydroxyurea, and ischemic heart disease. He was also a hepatitis B carrier. The patient had a coronary artery bypass graft more than 30 years ago. He had a recent history of sphincterotomy and stone extraction from the common bile duct. He had spondylodegenerative changes of the cervical spine and spinal cord edema at the C3/C4 disc level. The patient presented with a mildly swollen right upper lip that had persisted for 1.5 months. Physical examination showed an erythematous plaque on the right upper lip extending to the nasolabial fold, as shown in Figure 1. Induration and nodules were felt under the plaque. No pain or discharge were present. No enlarged lymph nodes were present. All other systems were reviewed and were negative.

The patient underwent a complete blood count, which showed that white blood cell count, platelet count, and creatinine were high; hemoglobin and hematocrit were low. A summary of the test results is provided in Table 1. Additional immunohistochemistry markers were as follows: TTF-1 (thyroid transcription factor-1) was negative and CK20 (cytokeratin 20) was positive in the tumor cells. Ki67 (Kiel 67) showed a high proliferative index. A summary of the immunohistochemistry results is provided in Table 2.

**Figure 2:**
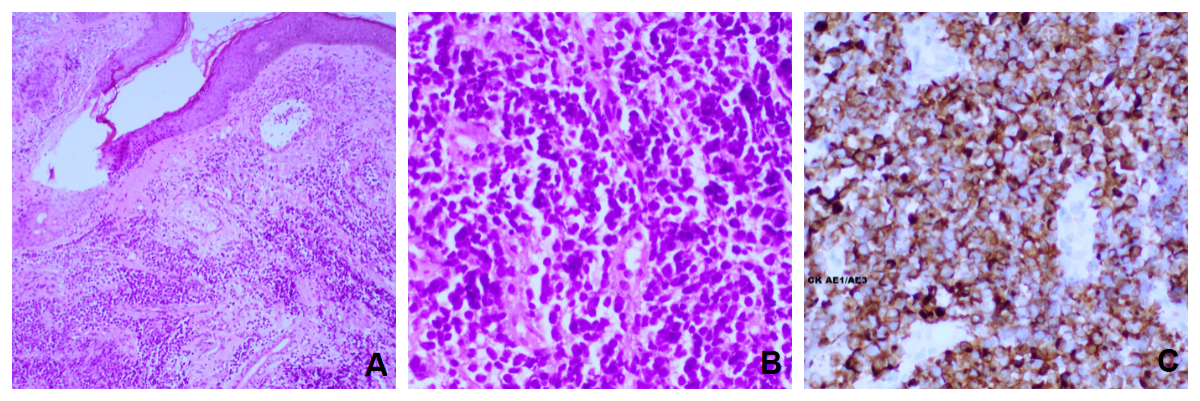
(A) Lower power hematoxylin and eosin staining revealed skin with diffusely infiltrative tumor within the dermis. Prominent solar elastosis and telangiectatic blood vessels are seen in the superficial dermis. (B) On higher magnification, tumor cells can be seen to be composed of small round blue cells with a high nucleus to cytoplasm ratio, round/oval hyperchromatic nuclei with a finely stippled salt and pepper chromatic pattern, indistinct nuclei, and scant cytoplasm. Mitoses and apoptotic bodies are seen in between. Nuclear molding and crush artifacts are noted. (C) Positive cytokeratin (AE 1/AE 3) with focal paranuclear staining.

Immunohistochemistry markers confirmed the diagnosis of MCC and ruled out a metastatic deposit of small cell carcinoma of the lung. Histopathological features were also in favor of MCC. They are represented in Figure 2.

The patient was referred to the oncology department for further management.

**Table 1:** The results of recent laboratory tests.

Name	Results	Normal range
White blood cells, n×10^3^/uL	16.4	3.6-11.0
Hemoglobin, g/dL	8.1	13.0-17.0
Hematocrit, %	25.2	40-50
Platelets, n×10^3^/uL	850	150-410
Lactate dehydrogenase, U/L	167	105-222
Creatinine, mg/dL	1.67	0.70-1.20

**Table 2:** Immunohistochemistry results.

Name	Results
Synaptophysin	Diffusely positive in tumor cells
Chromogranin	Diffusely positive in tumor cells
CD^a^56	Diffusely positive in tumor cells
Ki67^b^	Shows a high proliferative index
CK^c^ (AE 1/AE 3)	Positive with focal paranuclear staining
CD45	Negative in tumor cells
CD20	Negative in tumor cells
CD3	Negative in tumor cells
CD38	Negative in tumor cells
CD30	Negative in tumor cells
Melanin-A	Negative in tumor cells

^a^CD: cluster of differentiation.

^b^Ki67: Kiel 67.

^c^CK: cytokeratin.

## Discussion

MCC is a cutaneous neuroendocrine carcinoma that is aggressive and has a high tendency for metastasis. Because of the lack of symptoms, it is difficult to make an early diagnosis of MCC, which is often misinterpreted as a subcutaneous benign tumor, squamous cell carcinoma, or melanoma [5]. Some uncommon skin lesions, including MCC, require a high index of suspicion to be diagnosed. It is an uncommon and aggressive neuroendocrine skin tumor that accounts for fewer than 1% of all cutaneous malignancies. It often manifests as a red, purple, or violaceous firm, painless nodule or plaque. Because of its rarity, it is frequently confused with more common skin tumors [6]. The clinical differential diagnosis of MCC includes basal cell carcinoma, squamous cell carcinoma, melanoma, metastatic neuroendocrine carcinoma, lymphoma, and sebaceous carcinoma.

UV exposure and immunosuppression are the 2 primary etiological factors besides polyomavirus linked to an elevated risk of MCC. As determined by the UVB solar index, the regional incidence of MCC increases with increasing sun exposure. Most MCC cases are found in the skull, face, and neck regions, which are the most sun-exposed parts of the body [7]. Furthermore, many people who are diagnosed with MCC have a history of other sun-induced skin malignancies. Patients with suppressed or disordered immunity, such as those on immunosuppressive therapy for organ transplantation, hepatitis, or HIV infection, or those with B-cell malignancies such as multiple myeloma, non-Hodgkin lymphoma, and chronic lymphocytic leukemia, have a higher incidence of MCC [8]. Another similar case was reported in 2023 in an immunocompromised patient with diabetes and hepatitis B, suggesting that decreased immune surveillance in these patients results in increased viral replication and integration in the progenitor cells of MCC [9].

Surgical therapy is the foundation of treatment. It is normal practice to do a wide local excision with a clearance margin of 3 cm to 5 cm [10]. Lymph node dissection is generally recommended for patients with regional node metastases. In stage I and II illnesses, adjuvant radiation therapy is often suggested for the main site and lymph node basin. Chemotherapy is often reserved for patients with stage III illness [11]. Anti–programed cell death protein 1/anti–programed cell death ligand 1 (anti-PD-1/PD-L1) blocking immunotherapeutic drugs, such as pembrolizumab and avelumab, when administered as first-line treatment, lead to an objective response (ie, a partial response or a complete response) in as many as 50% to 70% of cases, making immunotherapy a promising new therapeutic option for advanced MCC [12]. Prompt management and treatment of MCC is essential because if left untreated, it can spread to other parts of the body and can also metastasize to lymph nodes and other organs [13].

## Conclusion

MCC is distinguished by violaceous, red intradermal nodules in sun-exposed locations. MCC of the skin is an aggressive lesion with a high risk of metastasis and recurrence; long-term (5-year) survival rates range from 18% to 57% [14]. The primary goal of presenting this case is to bring MCC, which is a diagnostic challenge, to the notice of dermatologists and oncologists, as early detection and prompt treatment are important.

## Abbreviations

Anti-PD-1/PD-L1: anti–programed cell death protein 1/anti–programed cell death ligand 1
CK20: cytokeratin 20
Ki67: Kiel 67
MCC: Merkel cell carcinoma
TTF-1: thyroid transcription factor-1
UV: ultraviolet
UVB: ultraviolet B
WBC: white blood cell
